# RNA Polymerase III Transcriptomes in Human Embryonic Stem Cells and Induced Pluripotent Stem Cells, and Relationships with Pluripotency Transcription Factors

**DOI:** 10.1371/journal.pone.0085648

**Published:** 2014-01-20

**Authors:** Ravi K. Alla, Bradley R. Cairns

**Affiliations:** 1 Department of Oncological Sciences, Huntsman Cancer Institute, University of Utah School of Medicine, Salt Lake City, Utah, United States of America; 2 Howard Hughes Medical Institute, Chevy Chase, Maryland, United States of America; University of Bristol, United Kingdom

## Abstract

Recent genomic approaches have revealed that the repertoire of RNA Pol III-transcribed genes varies in different human cell types, and that this variation is likely determined by a combination of the chromatin landscape, cell-specific DNA-binding transcription factors, and collaboration with RNA Pol II. Although much is known about this regulation in differentiated human cells, there is presently little understanding of this aspect of the Pol III system in human ES cells. Here, we determine the occupancy profiles of Pol III components in human H1 ES cells, and also induced pluripotent cells, and compare to known profiles of chromatin, transcription factors, and RNA expression. We find a relatively large fraction of the Pol III repertoire occupied in human embryonic stem cells (hESCs) and induced pluripotent stem cells (iPSCs). In ES cells we find clear correlations between Pol III occupancy and active chromatin. Interestingly, we find a highly significant fraction of Pol III-occupied genes with adjacent binding events by pluripotency factors in ES cells, especially NANOG. Notably, in human ES cells we find H3K27me3 adjacent to but not overlapping many active Pol III loci. We observe in all such cases, a peak of H3K4me3 and/or RNA Pol II, between the H3K27me3 and Pol III binding peaks, suggesting that H3K4me3 and Pol II activity may “insulate” Pol III from neighboring repressive H3K27me3. Further, we find iPSCs have a larger Pol III repertoire than their precursors. Finally, the active Pol III genome in iPSCs is not completely reprogrammed to a hESC like state and partially retains the transcriptional repertoire of the precursor. Together, our correlative results are consistent with Pol III binding and activity in human ES cells being enabled by active/permissive chromatin that is shaped in part by the pluripotency network of transcription factors and RNA Pol II activity.

## Introduction

Nuclear transcription is carried out by three distinct RNA Polymerases: RNA Polymerase I (Pol I), RNA Polymerase II (Pol II) and RNA Polymerase III (Pol III). Pol I transcribes a single long ribosomal RNA (pre-rRNA) transcript which is processed into 28S, 5.8S and 18S rRNAs [1]. Pol II transcribes primarily messenger RNA (mRNA) that code for proteins, as well as a variety of non-coding RNAs (ncRNAs), small nuclear RNAs (snRNAs), small nucleolar RNAs (snoRNAs) and micro RNAs (miRNAs) that are involved in gene regulation, RNA processing and chromatin organization. Pol III transcribes ncRNAs [1] that are mostly involved in translation, such as 5S ribosomal RNA, RNase P, RNase MRP and tRNAs. In addition Pol III also transcribes U6 RNA (involved in splicing); VA­I and VA­II (viral RNAs); Alu and MIR repeats (SINE elements); BC200 (involved in neuronal disease and messenger RNA translation; 7SK and BC2 (involved in Pol II transcription); BC1 (involved in spermatogenesis); Vault RNA (implicated multidrug resistance) and Y RNA (component of the Ro ribonuclear protein complex) [2].

**Figure 1:**
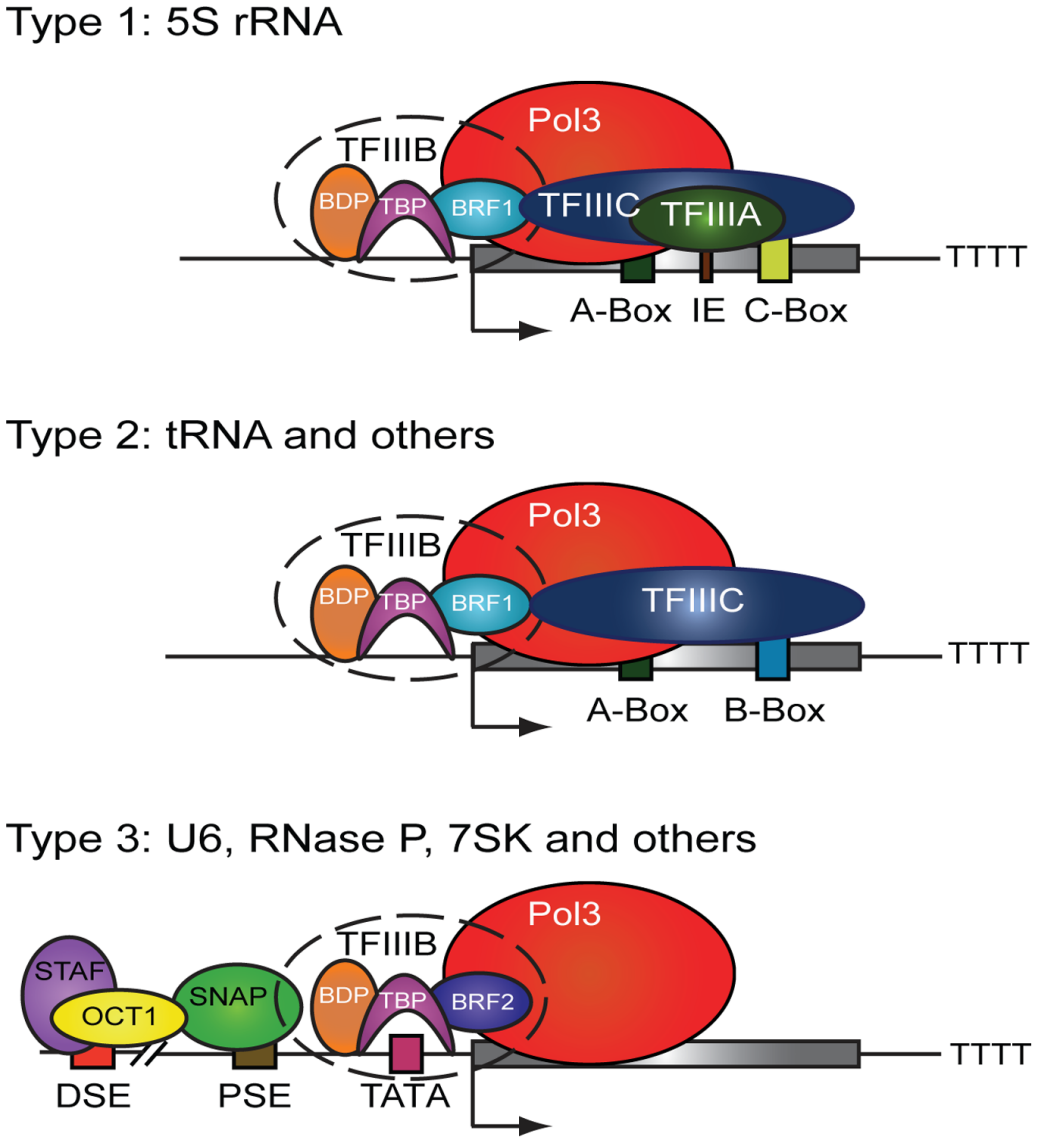
Pol III gene classes based on promoter architecture and associated transcription factors. IE – Internal Element, DSE – Distal Sequence Element, PSE – Proximal Sequence Element. Adapted from Ref 5.

Pol III transcription requires the transcription factor TFIIIB which helps recruit Pol III to its target genes and to initiate transcription. TFIIIB is composed of the TATA binding protein TBP, BDP1 and either BRF1 or BRF2. Pol III genes can be classified into three types based on their promoter structure, associated transcription factors and the composition of the TFIIIB complex [3] (**Figure 1**). Type I genes have an internal promoter comprised of A and C box sequences to which TFIIIA binds and recruits TFIIIC and the BRF1-containing TFIIIB to initiate Pol III transcription. The only Type I Pol III genes are the 5S rRNA genes. All the tRNAs genes fall in to the Type II category of Pol III genes. These genes have internal A and B box promoter sequences that are bound by TFIIIC (without the need for TFIIIA), and utilize a TFIIIB complex-containing Brf1, as do Type I genes. 7SL, BC200, vault RNAs and SINE elements (Alus and MIRs) constitute the remaining Type II genes. Type III genes in contrast are more similar to canonical Pol II genes in that they have a promoter sequence that is upstream of the transcription start site (TSS). The Type III gene promoter is made up of distal and proximal sequence elements (DSE and, PSE, respectively) and a TATA box. SNAPc binds the PSE, OCT1 binds the DSE and TFIIIB-containing BRF2 (instead of BRF1) binds the TATA box to help Pol III transcribe. This class of Pol III genes does not require TFIIIC for transcription [4]. 7SK, U6, RNase P, RNase MRP and the Y RNAs fall into this category. Notably most Pol III genes are less than 200 bp and are terminated by a sequence of thymidine residues.

The identification of active genes that are transcribed by Pol III has been traditionally difficult due to their repetitive nature and high copy number in most eukaryotic genomes. Assaying for the presence of a Pol III transcript does not identify the specific gene copy within the genome from which the transcript originated. However, recent studies used genome wide ChIP-seq approaches to identify Pol III binding sites in the human genome [5]–[9]. This approach involves the sequencing of unique flanking regions, enabling identification of Pol III-occupied regions. Here, the binding of Pol III and associated transcription factors was used as a proxy for active transcription. These studies, conducted in multiple cell types, identified all the previously-known Pol III transcripts in addition to several novel transcripts. Interestingly, only a fraction of the 513 *in silico* predicted tDNAs (tRNA genes) were occupied by Pol III, TFIIIC and Brf1 in each cell type. This is in stark contrast to budding yeast, where all the tDNAs are constitutively bound by Pol III [10]–[12]. This partitioning of Pol III binding is not explained by differences in promoter sequences of the tDNAs as the A and B-boxes are virtually identical in bound and unbound tDNAs, but instead depends on their location in the human genome. Active tDNAs are always found in regions of active chromatin in each cell type. About 20% of these active tDNAs are in promoters of known Pol II genes, and as expected have high levels of H3K4me3 and Pol II. However, 80% of the active tDNAs are found outside known Pol II promoters, but still reside in euchromatic regions. We previously classified these regions as enhancer-like regions, as they are marked by enhancer marks such as H3K4me1 and H3K27ac, and coincide with the binding of activating transcription factors such as ETS1, CBP, c-JUN, c-FOS, c-MYC and STAT1. Interestingly, these enhancer-like loci also bear similarity to promoters, as they also contain H3K4me3, and RNA Pol II, which in many cases creates a small RNA transcript in the opposite orientation to, and ∼300–400 bp away from, the active tDNA. We and others interpret this data as the formation of an initiation-competent Pol II enhancer/promoter that creates an open chromatin structure upstream and helps promote access of the Pol III machinery to the tDNA [5]–[8]. Inhibition of Pol II activity by low doses of α-amanitin can reduce expression of Pol III target genes. However, reduction of Pol II activity might affect many processes that indirectly lead to Pol III transcriptional attenuation [6], [8]. Notably, there are somewhat different repertoires as well as different number of Pol III-bound genes in different cell types. Thus far, all cells share a fraction of active tDNAs, those tDNAs located just upstream of housekeeping genes [5] but there are also cell type-specific tDNAs. Current models support the notion that cell specificity is conferred by unique chromatin landscapes imposed by different pools of transcription factors and that these, in turn, provide Pol III machinery access to different repertoires of its target genes.

To further understand the logic for defining the cell type-specific repertoire of Pol III, we chose a cell type of high interest, and with a relatively “open” chromatin status – human ES cells. Many studies are consistent with the notion that the open chromatin of ES cells enables them to differentiate into multiple different cell types during development [13]–[15]. ES cells show loose binding of HP1 proteins to the chromatin, rendering it more accessible to transcription factors and chromatin modifiers. Upon differentiation, HP1 begins to stably associate with chromatin [16]. Furthermore, studies comparing chromatin marks in hESCs and differentiated cell types show that ES cells have more H3K4me3 and H4ac [13], [15]. They also have lower levels of inactive marks such as H3K9me3 and H3K9me2, which become more enriched on differentiation [13], [17], [18]. ES cells are also characterized by bivalent domains that have both active (H3K4me3) and repressive (H3K27me3) marks [19]. These regions are thus ‘poised’ for activation or repression upon differentiation. ES cells also have a core pluripotency transcription factor network, that through feedback loops maintain an open chromatin state. The Pol III repertoire in mouse ES cells was previously studied but did not address the relationship of pluripotency transcription factors or chromatin dynamics to Pol III occupancy [20]. Our study aims to understand the relationship of human ES cell chromatin and transcription factors, especially pluripotency factors with Pol III occupancy. Here, we used publicly-available hESC chromatin marks and transcription factor ChIP-seq data **(Table S1** in **File S1**) to compare with our own genome wide Pol III and TFIIIC maps. We also used an induced pluripotent stem cell (iPSC) model to understand how the transition from a differentiated to pluripotent state affects Pol III binding.

## Materials and Methods

### Cell Culture and Cross-linking

H1 ES cells were obtained commercially from WiCell (H1-OCT4-EGFP). ADS-iPS cells were derived previously [21] at the Salk Institute and were a gift from Dr. Ron Evans. ADS cells were obtained from Invitrogen (Cat# R7788110). H1 and ADS-iPS cells were cultured using a feeder free system, with Matrigel (BD Biosciences) and mTESR medium (StemCell Technologies). ADS cells were cultured to 80% confluence according to manufacturers protocol (in MesenPRO RS medium) and cross-linked. The ES cells were expanded onto 100 mm matrigel coated plates and grown for 5 days (till optimal colony size was achieved) before cross-linking for ChIP. Cross-linking was performed with 1% (v/v) formaldehyde for 20 minutes followed by a 10 minute quenching in 125 mM glycine.

### Chromatin Immunoprecipitation

ChIP was performed as previously described [5]. Briefly, nuclei were lysed in 50 mM Tris-HCl pH 8, 100 mM NaCl, 10 mM EDTA and 1% (w/v) SDS. Chromatin was prepared by sonicating the nuclei (Misonix) 10–15 times on setting 4–5, to yield 200–400 bp fragments. The chromatin extract was pre-cleared with 50 ul Dynabeads (Invitrogen) in dilution buffer (15 mM Tris-HCl pH 8, 150 mM NaCl, 2 mM EDTA, 1% w/v Triton-X, 2 mg/ml BSA) for 1 hour. Immunoprecipitation was performed in dilution buffer, using 5–10 ug antibody conjugated to 100 ul Dynabeads, for 16 hours. The beads were then washed and eluted with 200 mM NaCl, followed by phenol:chloroform-isoamyl (25∶24∶1 pH 8, Invitrogen) extraction, ethanol precipitation and Qiagen MinElute purification. For Pol III ChIP, custom antibody against Rpc155 (generously provided by Dr. Robert White) was used and for TFIIIC, polyclonal antibody against TFIIIC63 from Bethyl laboratories (Cat# A301-242A) was used.

### Sequencing

Pol III and TFIIIC ChIP eluates were processed for sequencing using the Illumina Truseq library preparation kit. H1 Pol III and matching input sequencing was performed on Illumina GAII using 36 cycle runs. H1 TFIIIC, ADS Pol III, ADS input, ADS-iPS Pol III and ADS-iPS input sequencing runs were performed on Illumina HiSeq 2000 using 50 cycle runs.

### Processing and Analysis of ChIP-seq and Small RNA Data

Illumina generated fastq files were aligned to human genome build hg18 using either the bowtie (http://bowtie-bio.sourceforge.net) or novoalign(http://www.novocraft.com) aligners. H1 Pol III data was uniquely aligned using the ELAND aligner. ADS and ADS-iPSC Pol III data was uniquely aligned to hg18 using bowtie with parameters -a -m 1 -v 1–best –strata. For TFIIIC the parameters -a -m 3 -v 1–best –strata were used. To align to 5S and SNARs bowtie was run with parameters -k 15 -v 2–best –strata. Publically available histone and transcription factor data for H1 cells (**Table S1** in **File S1**) was aligned uniquely using bowtie (-a -m 1 -v 1–best –strata). The H1 (SRR020285) and H9 (SRR531470) small RNA-seq dataset was aligned to hg18 using novoalign (-a -r All 10). The resulting SAM alignment files were processed through the USeq [22] pipeline as described before. Briefly, the input and eluate alignments were converted to center position binary point data (SamParser). The point data was then filtered for duplicate alignments (PointDataManipulator), followed by the ScanSeqs program which uses a sliding window to compute smoothed window statistics. Finally, the ScanSeqs output was piped into EnrichedRegionMaker that combines windows based on user thresholds to output a list of enriched regions. ScanSeqs generated QValFDR tracks were used for visualization in IGB [23] (http://bioviz.org/igb/). For the small RNA-seq alignments, point data was generated as above and the read coverage track was generated (ReadCoverage) for visualization on IGB. Genome snapshots were obtained from IGB genome browser. To perform Venn intersections for enriched genes, enriched regions were intersected with known annotated Pol III gene bed file (**Table S2** in **File S1**). This file contains all known Pol III genes and all the Alus and MIRs from the hg18 repeat masker track from the UCSC browser. Once enriched regions were converted to enriched genes, Venn intersections were performed using a Venn diagram generator (http://www.pangloss.com/seidel/Protocols/venn.cgi Chris Seidel). To perform Venn intersections of enriched regions (instead of genes like above), pybedtools [24], [25] was used. The IntersectRegions program from USeq was used with enriched regions to perform intersection analysis to compare Pol III to chromatin marks and factors **(Table S9** in **File S1)**. Enriched region files were generated as above and the USeq program AggregatePlotter was used with point data of either the chromatin marks or transcription factors, to generate class average data. Prism (http://www.graphpad.com/scientific-software/prism/) was used to plot the class average graphs. For analyzing H3K27me3 levels at a region, USeq program DefinedRegionScanSeqs was used in conjunction with the chromatin mark’s point data and the corresponding region file. Prism was then used to plot log2enrichment values as box plots and for performing t-test to assess significance.

### Real-time PCR

For qPCR 1∶100 of the ChIP eluate was used with 500 nM primer mix and iQ SYBR Green Supermix (Bio-rad) in a total volume of 25 ul. Serial dilutions were performed with corresponding input DNA for the standard curve. PCR cycling was performed according to standard protocols with an annealing temperature of 62–65C on a CFX96 (Bio-rad) system. Results were analyzed using the iCycler (Bio-rad) program and excel. Primers used for analyzing ChIP efficiency can be found elsewhere [5].

## Results

### Localization of Pol III and TFIIIC in Human Embryonic Stem Cells

**Figure 2:**
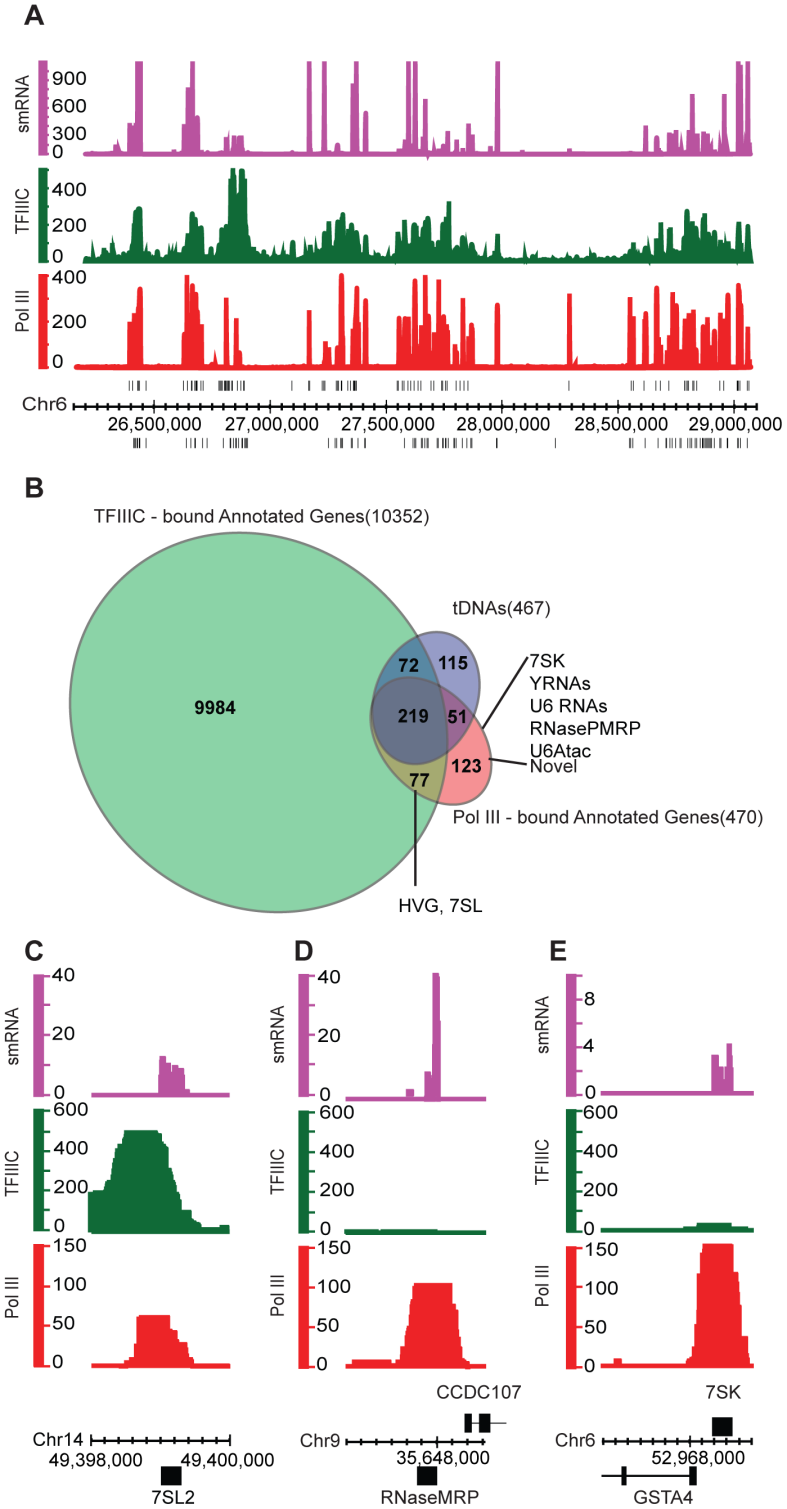
Occupancy analysis of Pol III and TFIIIC in H1 cells. **A.** Chromosome 6 tDNA cluster snapshot showing Pol III QValFDR (red), TFIIIC QValFDR (green) and the H1 small RNA read coverage (magenta) tracks. Chromosome coordinates are shown under the plots, tDNAs are depicted as black vertical lines. Annotations above the chromosome coordinate line (x-axis) are on the (+) strand and those below are on the (−) strand. The y-axis values are −10Log(QValue FDR) scores. **B.** Venn diagram depicting intersection between mappable tDNAs (blue), Annotated Pol III genes bound by Pol III in H1 cells (red) and Annotated Pol III genes bound by TFIIIC in H1 cells (green). Numbers in parentheses indicate the number of genes in each Venn set. **C–E.** Pol III QValFDR, TFIIIC QValFDR and small RNA read coverage tracks at Pol III example loci. 7SL2 is a Type II Pol III gene (**C**); RNAseMRP (**D**) and 7SK (**E**) are Type III Pol III genes.

To understand the Pol III repertoire and localization in a pluripotent landscape, chromatin immunoprecipitation (ChIP) was performed in H1 human embryonic stem cells. Here, we utilized antibodies against Rpc155 (Pol III subunit) and TFIIIC63 (TFIIIC subunit). Initial ChIP efficiency was validated by quantitative PCR, at known Pol III and TFIIIC target regions (**Figure S1A,B**). High-throughput sequencing of the ChIP eluate (ChIP-Seq) was then performed to obtain genome-wide Pol III and TFIIIC occupancies in H1 cells. Here, we can attribute Pol III binding within the genome, even at most repetitive regions and to tDNAs, because of unique sequences present in their flanking regions. We then aimed to link Pol III occupancy to transcript generation. However, many Pol III transcripts are generated from non-unique or repetitive loci (tDNAs, Alu elements), and in those cases the generated transcripts are identical. Allowing for the random partitioning of reads that align to multiple regions of the genome, one can visualize RNA at repeat regions in a ‘class average’ manner. Here, the small RNA dataset from H1 ES cells was aligned to the human genome, allowing random partitioning of reads from repeat sequences, providing signal in the small RNA track that for non-unique/repetitive loci (as a ‘class average’) might not always represent an actual transcript from that location. We then examined the tDNA cluster on human chromosome 6, which shows occupancy by Pol III, TFIIIC and the presence of small RNA reads (10–50 bp sized RNA)(**Figure 2A**). To identify high-confidence Pol III- and TFIIIC-bound regions in the human genome, false discovery thresholds (FDR) of 1% and 0.01% were set for Pol III and TFIIIC respectively. We chose a more stringent threshold for TFIIIC data to limit the enriched regions to a computationally manageable number (Note: TFIIIC is known to bind tRNAs and highly abundant Alu/MIR repeat elements in other cell types [5]). Using these thresholds, we obtained 330 Pol III-bound regions and 8926 TFIIIC-bound regions. These regions were intersected with a list of annotated Pol III genes, containing all known Pol III genes and all the Alus/MIRs identified using the ‘repeat masker’ table present on the UCSC hg18 genome repository (**Table S2** in **File S1**). This yielded a total of 470 Pol III-bound genes and 10352 TFIIIC-bound genes. There are more factor-bound genes than regions because certain regions are large enough to house multiple closely-spaced Pol III genes or Alu/MIR elements. Of the 467 mappable tDNAs in human cells [5], 270 were bound by Pol III in H1 cells and 219 were bound by both Pol III and TFIIIC (**Figure 2B** **,** **Table 1**). This observation conforms to studies by us and others in various human cell types [5]–[8], which show that only ∼50% of all tDNAs are bound by Pol III. In H1 cells this fraction of Pol III-bound tDNAs is higher at 58%. 51 of the Pol III-bound tDNAs in H1 cells are scored as not bound by TFIIIC (FDR 0.1%); however, more than half of these Pol III-only tDNAs are scored as bound by TFIIIC at a lower FDR threshold (1%). In addition to tDNAs, Pol III also bound Type I (5S rRNA genes, **Table 1**), other Type II (**Figure 2C**) and Type III genes (**Figure 2D,E**). As expected Type III genes (7SK, RNase MRP) were not bound by TFIIIC (**Figure 2D,E**). 5S and snaR genes (Type II) are non unique and were analyzed by allowing for multiple matches during alignment. In addition to annotated genes, Pol III also bound repeat elements: Alu (106 bound), Alu monomers (FLAM/FRAM repeats, 41 bound) and MIR repeats (25 bound). We provide a description of the known annotated Pol III genes bound by Pol III in H1 cells (**Table 1**), the full list of Pol III-bound regions, and the top 500 TFIIIC-bound regions (**Tables S3, S4** in **File S1**).

**Table 1:** Annotated Pol III genes in the hg18 human genome and Pol III enrichment in H1, ADS-iPS, ADS and HeLa cells.

Cell Type		H1				ADS-iPS	ADS	HeLa
Promoter Type	ncRNA	# in humangenome	number boundby Pol III	#bound by Pol III and TFIIIC	#bound by TFIIIC only			
Type I	5S	17	17	17	17	17	17	17
Type II	tRNAs	513	272	219	98	329	244	225
	tRNA-pseudogene	172	6	2	19	9	2	2
	Alu-dimer	1,099,242	106	63	7563	85	46	36
	Alu-monomer	90,882	41	0	1762	4	2	4
	MIR	587,443	25	9	4424	18	7	12
	HVG	3	2	2	1	2	3	3
	7SL	2	2	2	0	2	2	1
	BC200	1	0	0	0	0	0	1^A^
	snaR	21	21	21	0	21	21	21
Type III	Y	4	4	NA	NA	4	4	4
	U6	9	4	NA	NA	4	4	4
	7SK	1	1	NA	NA	1	1	1
	RNaseP	1	1^B^	NA	NA	1	1	1
	RnaseMRP	1	1	NA	NA	1	1	1
	U6Atac	1	1	NA	NA	1	1	1
	tRNASec	3	1	NA	NA	1	1	1

A - Detected only in Pol III ChIP-chip experiments.

B - Detected by ChIP-qPCR (Figure S2).

### The Pol III Repertoire in H1 Cells Compared to Other Human Cell Types

**Figure 3:**
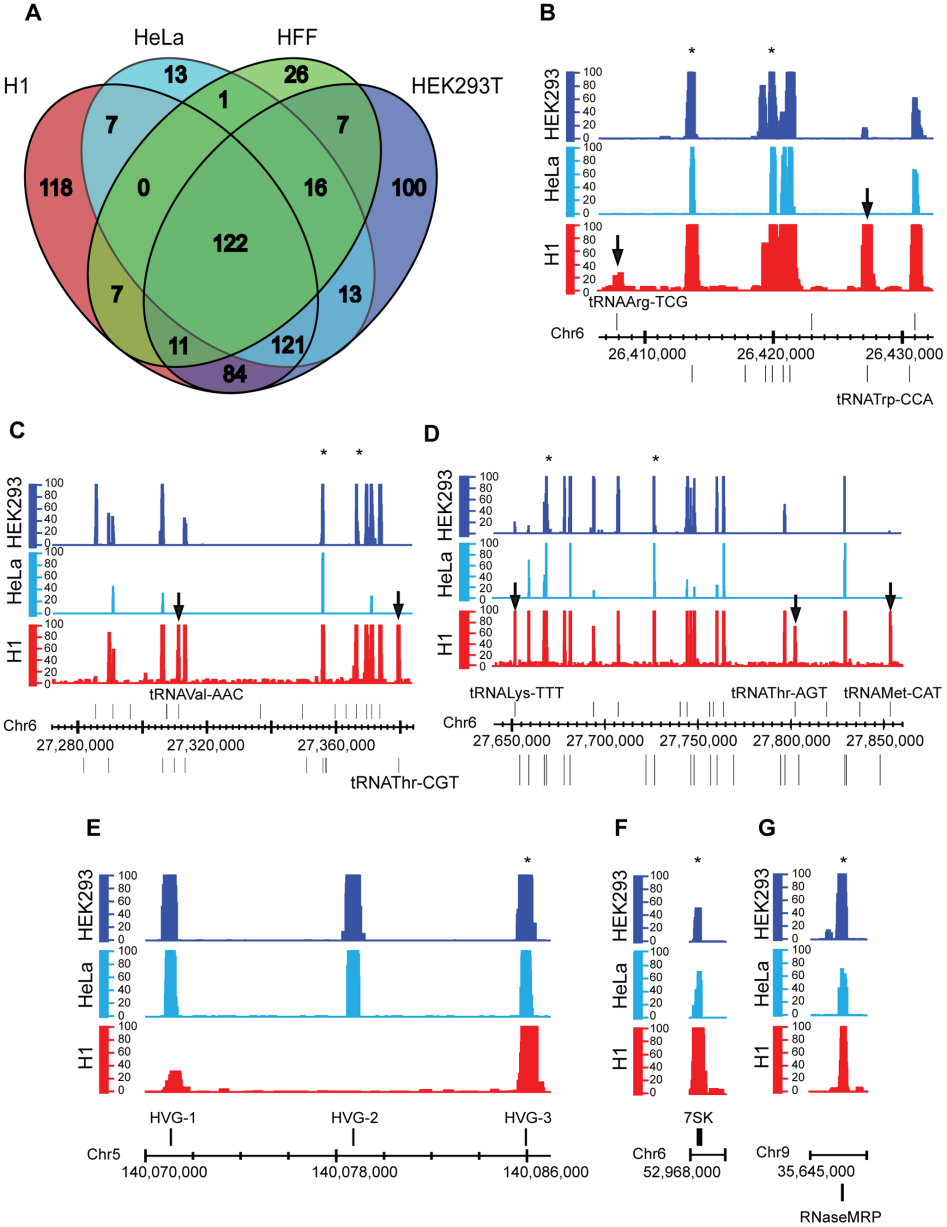
Differential occupancy by Pol III in various cell types. **A.** Venn diagram showing intersection of Pol III-bound annotated genes in four different cell types, H1 (red), HeLa (cyan), Human Foreskin Fibroblasts - HFF (green) and HEK293T cells (blue). **B–D.** Pol III QValFDR tracks at example tDNAs that are differentially bound in H1 cells (red) compared to HeLa (cyan) and HEK293 (blue) cells. Arrows mark the genes that differ in Pol III occupancy. Asterisks mark the tDNAs that are similarly occupied in all the cell types. The axes are as described in Figure 1. **E–G.** Pol III QValFDR tracks at example Type II (**E**) and Type III (**F,**G) loci that are similarly bound in the three cell types. Asterisks mark the genes that are bound by Pol III in all cell types.

Embryonic stem cells have been studied extensively, and are widely believed to possess chromatin that is more open and active than somatic/differentiated cells [13]. Therefore, we wanted to compare the Pol III repertoire in H1 cells with previous analyses in other cell types. A list of Pol III-bound regions (FDR 1%) in H1, HEK293T, human foreskin fibroblasts (HFF) and HeLa was defined, and then intersected with a list of annotated Pol III genes (**Table S2** in **File S1**). We find 470 occupied, annotated Pol III genes in H1 cells, 467 in HEK293T cells, 190 in HFF cells and 293 in HeLa cells (**Figure 3A**). Examination of the datasets strongly suggests that these cell type-specific differences in the number of Pol III-bound genes are not simply due to FDR thresholds. For example, we see clear instances of Pol III genes that are bound in H1 and not in HeLa and HEK293 cells; certain tDNAs are bound at high levels in H1 cells (**Figures 3B–D** **,** shown with arrows), but not in the other cell types, and may be compared to neighboring tDNAs that are roughly equally bound in all the cell types examined (**Figures 3B–D** **,** selected examples marked with asterisks). We also see examples of other types of Pol III genes that are bound in all three cell types (**Figures 3E–G**). Interestingly, we find more Pol III-bound genes in H1 cells than in cancer (HeLa) or differentiated cells (HFF). The number of Pol III-bound genes is comparable between HEK and H1 cells, which may be due to the embryonic origin of the HEK293T cells. There is also a large set (122) of Pol III-bound genes that are common to all four cell types, 25% of which are in known Pol II promoters. Further, there are 100 Pol III genes that are unique to HEK cells and 118 that are unique to H1 cells (**Table S5** in **File S1)**, compared to only 26 and 13 genes in HFF and HeLa respectively (**Figure 3A**). The possible relationship of pluripotency network factors with these H1 specific Pol III genes is explored later in the paper.

### Pol III Binds Genes in Active Chromatin

To assess the relationship between Pol III binding and active chromatin [5]–[9] in human ES cells, we utilized publicly available genome-wide chromatin data in conjunction with our own Pol III and TFIIIC data and reprocessed all the data through our pipeline. Here, our analyses will focus on tDNAs, as they represent the largest category (513 tDNAs) of Pol III-transcribed loci and show cell type-specific variance in Pol III occupancy, whereas there is no such variation at Type I and Type III genes. To compare Pol III occupancy to chromatin features, we first binned tDNAs into four categories: Top 50 (blue), Middle 50 (red), Bottom 50 (green) and ‘unbound’ (cyan), based on their Pol III occupancy (FDR 1%). The ‘unbound’ category contains tDNAs that have no Pol III or TFIIIC and serve as a negative control in the analyses.

**Figure 4:**
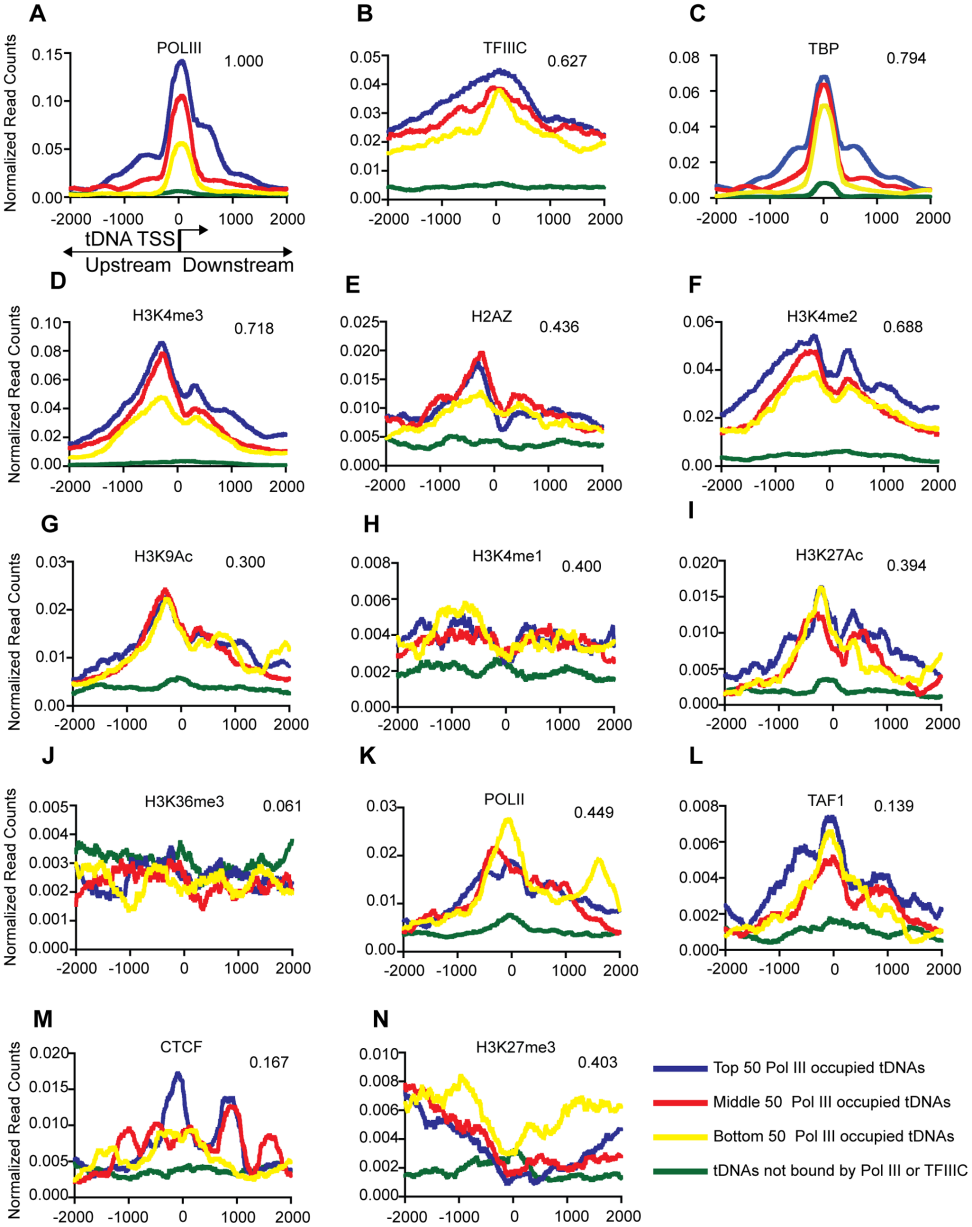
Chromatin marks and factors associated with Pol III-bound genes in H1 cells. Class average maps of histone marks and transcription factors at a 4-axis shows the distance from the TSS of the Pol III gene (0 is the start of the TSS). **Figure 4A** shows the schematic of a tDNA along the x-axis. The y-axis shows normalized read counts. Note that the scale of the y-axis is different for the various factors and marks. tDNAs were binned based on levels of Pol III binding, top 50 tDNAs (blue), middle 50 tDNAs (red), bottom 50 tDNAs (yellow) and tDNAs not bound by Pol III or TFIIIC (green). Also shown are the intersection fractions of Pol III-bound regions with the mark/factor bound regions (top right of each graph). Intersections are significant with a P-value <0.001.

We then performed class average analysis, where the x-axis shows the distance from the transcription start site (TSS) of a tDNA and the y-axis shows normalized read counts for the specified factor (**Figures 4A–N**). The intersection fraction of the Pol III-bound genes with each factor is also shown on the graphs. This intersection fraction is the measure of overlap with a correlated p-value between Pol III binding and corresponding factor/mark. The 4 categories are indeed based on Pol III occupancy (**Figure 4A**), and the TFIIIC and TBP levels (**Figure 4B,C**) scale with Pol III occupancy as well. tDNAs bound by Pol III are highly correlated with the active chromatin marks H3K4me3, H2AZ, H3K4me2 and H3K9ac (**Figures 4D–G**) and show a peak of active marks upstream of the TSS. This trend is expected for tDNAs that reside within annotated Pol II promoters, but only 16% of all Pol III-bound tDNAs fall into this category. The other 84% of Pol III-bound tDNAs fall in areas of active chromatin we previously termed enhancer-like regions. In keeping with our previous observations, H3K4me1 and H3K27ac mark these enhancer-like regions, and are correlated with Pol III occupancy in H1 cells (**Figure 4H, I** **)**. The distribution of the H3K4me1 mark is subtle compared to other marks. It modestly peaks upstream of the Pol III TSS and intersection with Pol III-bound regions is quite high (40%, **Figure 4H**). Pol III-bound tDNAs are not correlated with H3K36me3 (correlated with repression of Pol II transcriptional initiation [26], **Figures 4J**). In contrast, active (Pol III-bound) tDNAs have a peak of Pol II upstream of their TSS **(** **Figure 4K** **)**. We also observed correlation between Pol III occupancy and the basal transcription factor TFIID subunit TAF1 (**Figure 4L**). In addition, active tDNAs correlated with CTCF binding sites (**Figure 4M**). Interestingly, unlike in HeLa cells, active tDNAs correlated with repressive H3K27me3 regions in H1 cells (**Figure 4N**). This regional correlation with H3K27me3 is further explored in the following section.

### Pol III-bound Genes in H1 Cells Correlate with Regional H3K27me3

**Figure 5:**
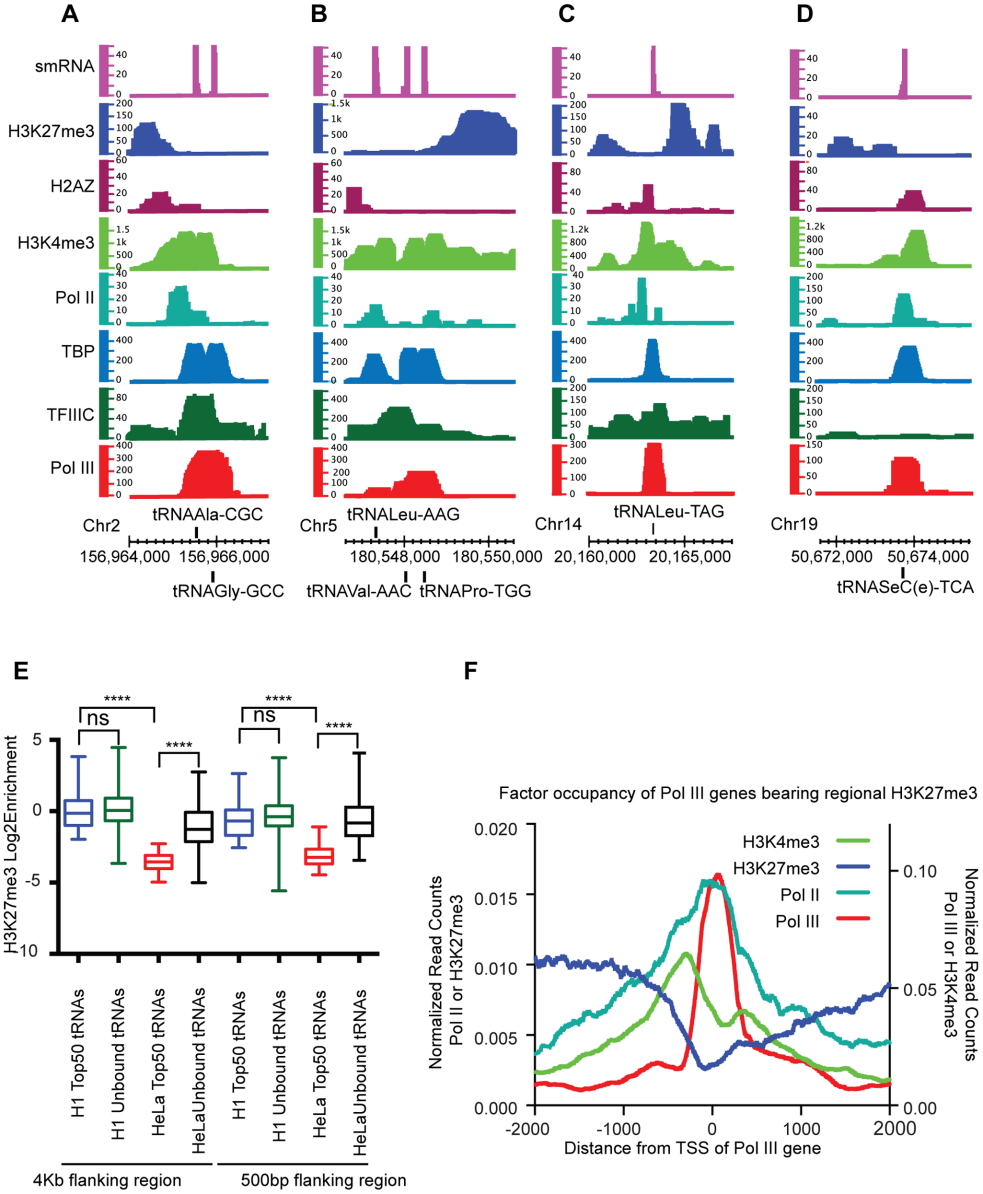
Analysis of Pol III-bound genes with regional H3K27me3. **A–D**. Examples are shown of Pol III-bound genes that intersect with H3K27me3 (within 2 kb). Shown are QValFDR tracks for Pol III (red), TFIIIC (dark green), TBP (cyan), Pol II (teal), H3K4me3 (light green), H2AZ (purple), H3K27me3 (blue) and the H1 small RNA read coverage track (magenta). Axes are as described in Figure 1. **E.** Box plot showing the difference in H3K27me3 enrichment levels between H1 and HeLa cells at Pol III-bound and unbound tDNAs (4 kb flanking TSS-left half of the plot and 500 bp flanking TSS-right half of the plot). Y-axis is the log of the H3K27me3 mark enrichment. Asterisks denote significant P-Value <0.0001. ns denotes a P-Value that is not significant. **F.** Class average map of H3K4me3 (green), H3K27me3 (blue), Pol II (teal) and Pol III (red) across an average Pol III-bound gene that is marked with H3K27me3. Axes are as described in Figure 3.

Pol III-bound regions are generally positively correlated with active chromatin marks and negatively correlated with silencing marks. Surprisingly, in H1 cells active tDNAs are correlated with windows (500 bp) containing the repressive mark H3K27me3 (FDR 1%), with a 40% intersection percent compared to 0% in HeLa cells [5] (**Figure 4N** **,** **Figure 5A–D**). In H1 cells, the top 50 Pol III-bound tDNAs have significantly higher levels of H3K27me3 compared to the top 50 tDNAs in HeLa cells (**Figure 5E** **, box plot**). Further, in HeLa cells there is a significant difference between H3K27me3 levels in bound tDNAs compared to unbound ones. This difference between bound and unbound tDNAs is no longer present in H1 cells. Closer inspection reveals that majority of the active tDNAs with H3K27me3 occur in the context of neighboring H3K4me3. In fact, 25% (68/270) of active tDNAs are in regions with H3K27me3 neighboring H3K4me3 whereas there are no active tDNAs in H3K27me3-only regions (those that have no H3K4me3). Interestingly, there are many examples of active (signal in the small RNA track) Pol III-bound genes, with nearby H3K27me3 but with H3K4me3 interspersed between the H3K27me3 and the Pol III peak (**Figure 5A–D**). This finding is supported by the observation that many active tDNAs are surrounded by H3K4me3 and Pol II. Class average maps show H3K27me3 often enriched ∼1 kb upstream of the Pol III gene TSS but falls off sharply at the TSS of the Pol III gene (**Figure 5F** **, Figure S2A**). Notably, H3K4me3 levels are inversely correlated with H3K27me3, they rise as H3K27me3 falls, and H3K4me3 levels peak ∼200 bp upstream of the Pol III TSS (**Figure 5F**). This is in contrast to a typical bivalent Pol II promoter where H3K4me3 and H3K27me3 are coincident around the TSS (**Figure S2B**). In addition, Pol II levels similarly peak at or adjacent to the Pol III TSS. Taken together, these results raise the possibility that Pol II-established active chromatin (high H3K4me3) insulates Pol III transcription from silencing from the adjacent H3K27me3 (**Figure 5A–D** **,** **Figure 5F**).

### Pluripotency Transcription Factors Localize to Pol III-bound Genes in H1 Cells

**Figure 6:**
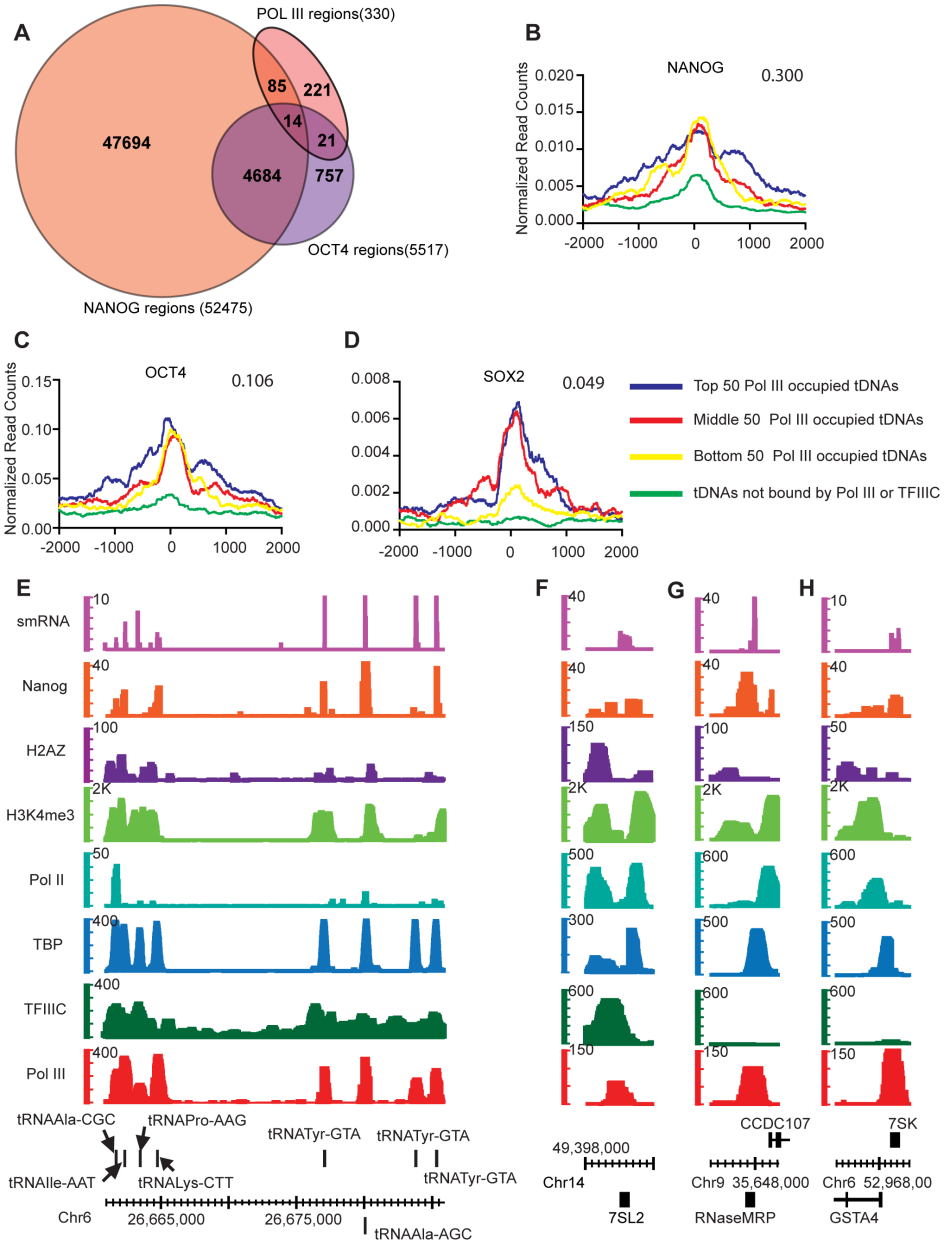
Occupancy analysis of pluripotency factors at Pol III-bound genes in H1 cells. **A.** Venn diagram showing intersection of Pol III-bound regions with Nanog and Oct4 bound regions in H1 cells. Numbers in parentheses indicate total number of regions bound by the factor. **B–D.** Class average maps of NANOG, OCT4 and SOX2 at a tDNA. Axes are as described in Figure 3. Also shown are the intersection fractions of Pol III-bound regions with the mark/factor bound regions (top right of each graph). Intersections are significant with a P-value <0.001. **E.** Examples of tDNAs that are bound by Pol III and Nanog. Shown are QValFDRs along the y-axis for Pol III (red), TFIIIC (dark green), TBP (cyan), Pol II (teal), H3K4me3 (light green), H2AZ (purple), H3K27me3 (blue), Nanog (orange) and the H1 small RNA read coverage (magenta) track. Axes are as described in Figure 1. **F–H.** Examples of Type II (7SL2) and Type III (RNaseMRP, 7SK) Pol III genes that are bound Pol III and Nanog.

**Figure 7:**
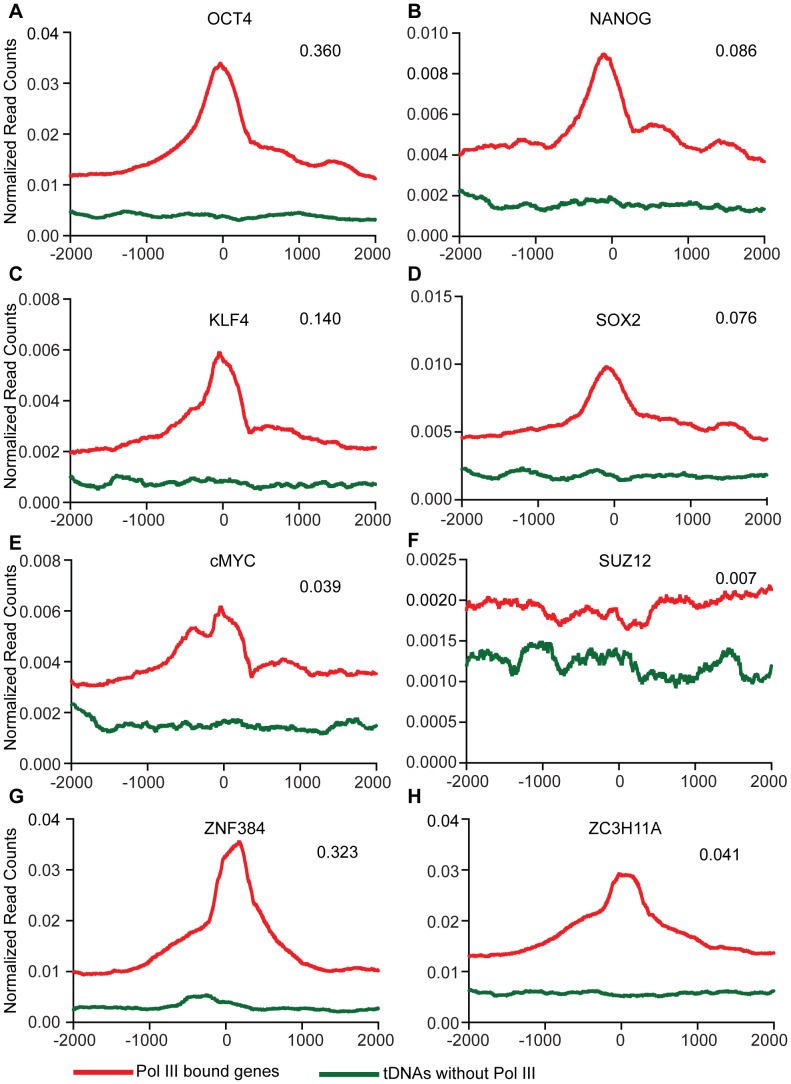
Pluripotency factors associated with Pol III-bound genes in mESCs. Class average maps of transcription factors at a 4-bound gene (red) or an average tDNA not bound by Pol III (blue) in mouse ES cells. Axes are as described in Figure 3. Also shown are the intersection fractions of Pol III-bound regions with the mark/factor bound regions (top right of each graph). Intersections are significant with a P-value <0.001.

Transcription factors normally associated with Pol II regulation such as STAT1, ETS1 and CBP co-localize to Pol III-bound regions in HeLa and Jurkat cells [5], leading to models that Pol III is able to occupy open chromatin created by binding of these and other transcription factors. To test this hypothesis in ES cells, Pol III occupancy in H1 cells was compared to available genome-wide maps for pluripotency factors. Remarkably Pol III-bound genes overlap with NANOG and OCT4 regions (**Figure 6A**). Pol III-bound genes correlated significantly with NANOG (30%, P<0.001), OCT4 (10%, P<0.001), and SOX2 (4.8%, P<0.001). NANOG, OCT4 and SOX2 localize around the TSS of tDNAs and scale positively with Pol III occupancy (**Figure 6B–D** **)**. NANOG occupies tDNAs and Type II/III genes including 7SL, RNaseMRP and 7SK (**Figure 6E–H**). 70 of the 99 Pol III and NANOG-bound regions, had consensus NANOG binding motifs within a 500 bp flanking region (P<0.0001 by FIMO [27] analysis). Next, the 118 H1 specific Pol III genes (**Figure 3A**) were analyzed to see if pluripotency factors bound them. We intersected the 118 H1 specific Pol III genes with NANOG-bound regions in H1 cells (FDR 1%). Of the 118 regions, NANOG bound 16, whereas OCT4 bound 42. We note that direct tests for a causative relationship were not possible due to ES cell differentiation and the resulting chromatin changes that accompany the knockdown of these factors [28]–[30]. Previous studies in mouse embryonic stem cells (mESCs) [20] examined Pol III occupancy but did not address the correlation with pluripotency factors. We obtained Pol III-bound regions from mESCs and correlated with available mouse NANOG, OCT4, and SOX2 data. In mESCs Pol III-bound genes correlate with OCT4 at 36%, NANOG at 8.6%, KLF4 at 14%, and SOX2 at 7.5% of regions (P<0.001). These transcription factors peak at or near the TSS of the Pol III gene (**Figure 7A–E**). As with H1 cells, transcription factor binding occurs only at Pol III genes that are bound by Pol III and not at tDNAs that are not bound by Pol III (green line on graphs **Figure 7A–E**). Suz12 is a repressive chromatin modifier and does not correlate with Pol III occupancy, thus serving as a negative control (**Figure 7F**). We also compared Pol III occupancy with other mESC transcription factor data (ZNF384 and ZC3H11A **Figures 7G–H**). Notably, the zinc finger transcription factor ZNF384 (also knowns as nuclear matrix protein 4 NMP4 and p130Cas interacting zinc finger protein, CIZ) intersects with 32% of Pol III-bound genes in mESCs (P<0.001 **Figure 7G**). Taken together, this correlative data raises the possibility that pluripotency transcription factors, and possibly ZNF384, play a key role in Pol III occupancy in embryonic stem cells.

### Induction of Pluripotency Affects the Pol III Repertoire

**Figure 8:**
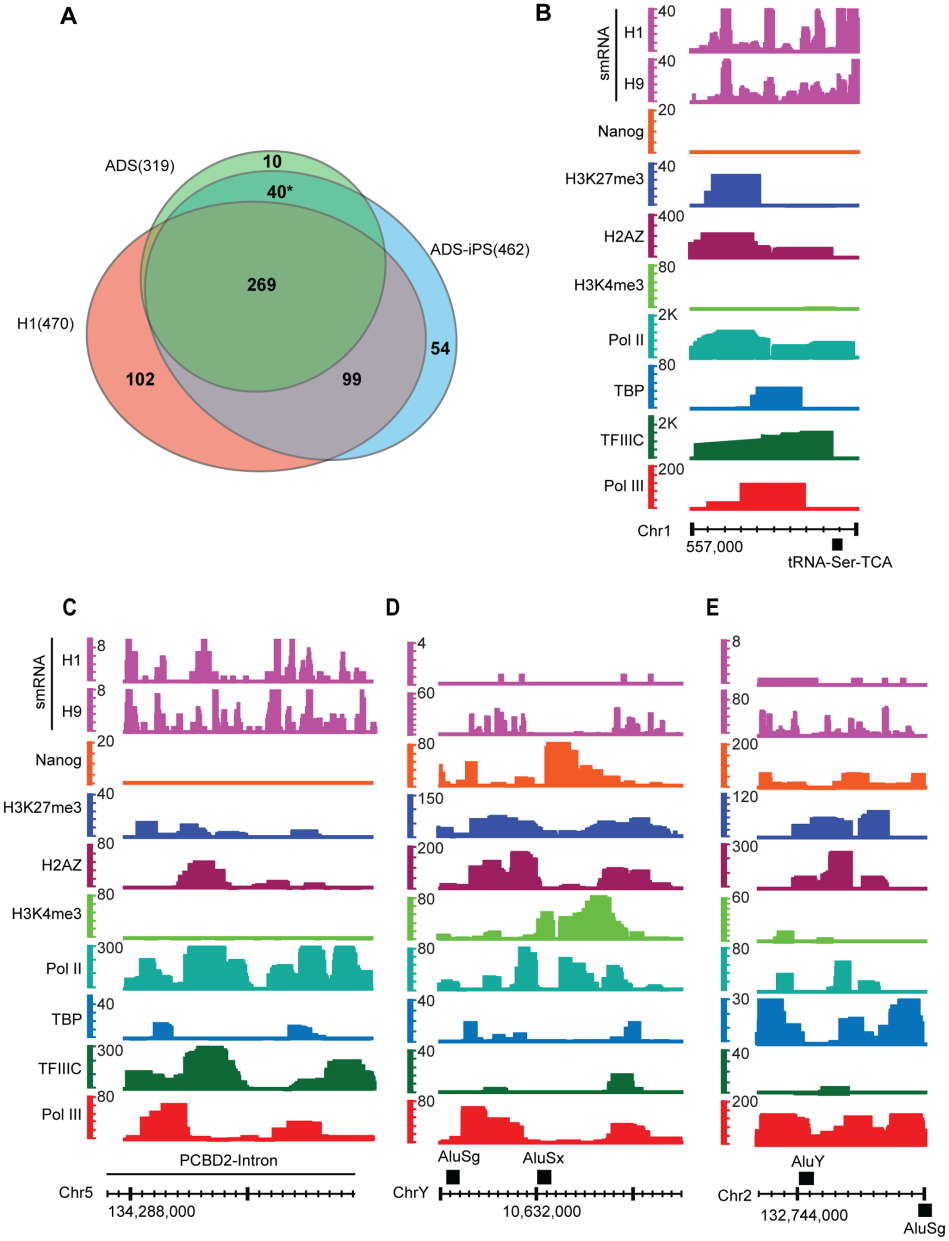
Pol III occupancy in precursor and iPS cells and novel Pol III-bound regions in H1 cells. **A.** Venn diagram showing intersection of Pol III-bound genes in H1, ADS and ADSiPS cells. Numbers in parentheses indicate number of Pol III-bound genes in each of the cell types. Asterisk indicates genes that are commonly bound in ADS and ADS-iPS cells but not in H1 cells. **B–E.** Examples of Novel Pol III-bound regions in H1 ES cells. Shown are QValFDR values along the y-axis, for Pol III (red), TFIIIC (dark green), TBP (cyan), Pol II (teal), H3K4me3 (light green), H2AZ (purple), H3K27me3 (blue), Nanog (orange) and H1 along with H9 small RNA read coverage (magenta).

To understand how changing the chromatin landscape back to a pluripotent state affects Pol III occupancy, genome-wide Pol III localization studies were performed in precursor (adipose derived stem cells ADSCs) and induced pluripotent stem cells (ADS-iPSCs). These ADS-iPSCs were derived from ADSCs using the four-factor (OCT4, KLF4, cMYC and SOX2) retroviral infection [21]. Upon induction of pluripotency significantly more Pol III genes were bound compared to the precursor cells, consistent with our observations in ES cells compared to highly differentiated cells. In ADSCs Pol III binds 312 genes compared to 455 genes in ADS-iPSCs (**Figure 8A** **)**. Here, 309 Pol III genes were bound in both cell types and only 10 Pol III genes are unique to ADSCs, compared to 153 in ADS-iPSCs. Furthermore, ADS-iPSCs had 329 tRNAs bound compared to only 244 tRNAs in ADSCs. Previous studies have shown that iPSCs are similar to ESCs by many criteria, but still retain chromatin, epigenetic and transcriptional features of their precursors [21], [31], [32]. This notion seems to hold true with respect to the Pol III transcriptome as well, as ADS-iPSCs and ADSCs share 40 Pol III-bound genes (**Figure 8A** **,** marked with an asterisk, P<0.001) that are not bound in H1 cells. We postulate that these 40 active Pol III genes are remnants of the ADSCs-active Pol III repertoire in the ADS-iPSCs that were not reprogrammed during the transition.

Because pluripotency factor maps are not available for ADS-iPSCs, the iPSC unique genes were compared to available H1 NANOG, OCT4 and SOX2 data. Of these 153 ADS-iPSC unique genes, 42 (27%, P<0.001) are bound by NANOG, 10 (6%, P<0.001) by OCT4 and 10 (6%, P<0.001) by SOX2 in H1 cells. **Table 1** shows the differences between the two cell types at all annotated Pol III genes. We also provide lists of Pol III-bound regions in both the precursor and iPS cells (**Tables S6, S7** in **File S1**).

### Novel Pol III Genes in Embryonic Stem Cells

To identify novel Pol III-bound genes, Pol III-bound regions in H1 and ADS-iPS cells were intersected with the list of known Pol III annotated genes (**Table S2** in **File S1**), and regions that failed to intersect were further inspected. 59 novel binding sites were identified for Pol III binding in H1 cells (**Table S8** in **File S1**), 15 of which have TFIIIC and 18 have small RNA. We show four examples of these novel Pol III-bound regions in H1 cells (**Figure 8B–E**). These loci have hallmarks of Pol III-bound regions, such as H3K4me3 and Pol II occupancy. These novel Pol III genes also show small RNA in H9 ES cells. These regions are not conserved among mammals (data not shown), which is not surprising, as many non-coding transcripts are not conserved across species at the sequence level. Small RNA sequencing and Pol III genome-wide data in other hESC and iPSC lines is needed to determine conservation of transcription and binding at these novel regions, before performing functional studies.

### TFIIIC-bound Regions in H1 Cells

**Figure 9:**
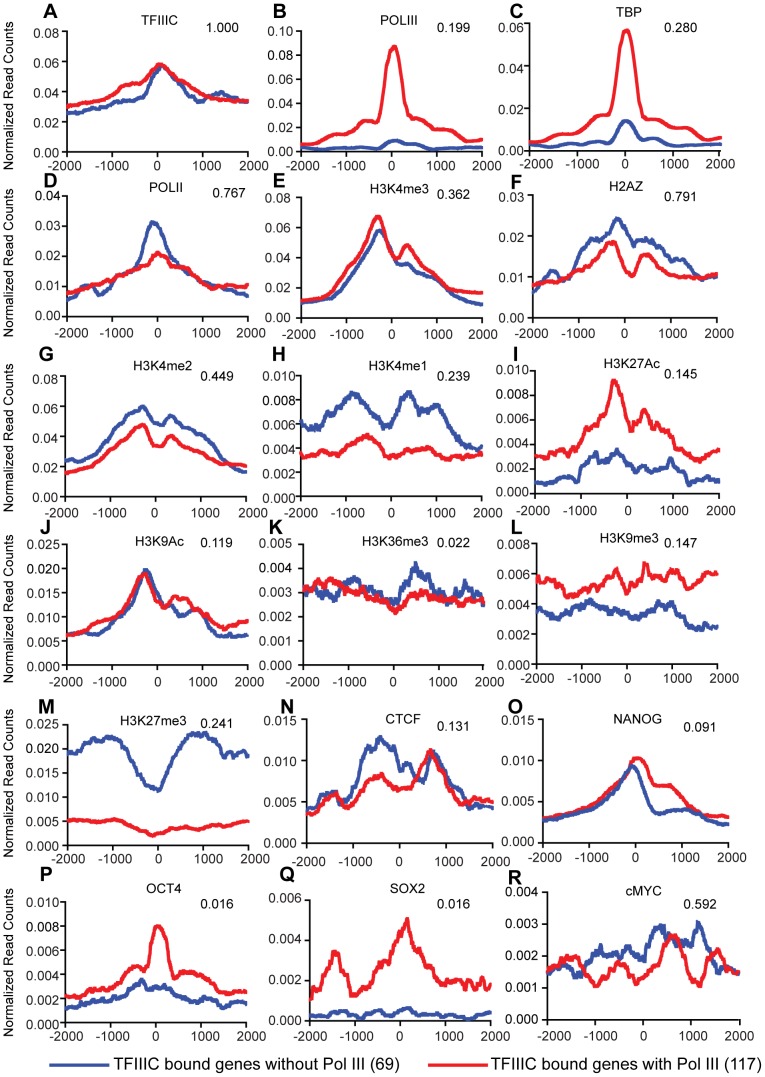
Chromatin marks and factors associated with TFIIIC-bound regions in H1 cells. Class average maps of histone marks and transcription factors at a 4-bound gene without Pol III (blue) or a TFIIIC-bound gene with Pol III (red). Axes are as described in Figure 3. Also shown are the intersection fractions of TFIIIC III bound regions with the mark/factor bound regions (top right of each graph). Intersections are significant with a P-value <0.001. The numbers in the parentheses next to the figure labels represent the number of regions interrogated.

TFIIIC-bound sites without Pol III (partially assembled Pol III transcription units) were shown to be important for chromatin organization in yeasts [33] and humans [7], [8]. These sites also correlate with areas of active chromatin [5] and CTCF binding [7], [20]. To perform similar analyses the top 500 TFIIIC-bound regions (**Table S4** in **File S1**) in H1 cells were analyzed with chromatin and transcription factor data. Class average maps were plotted for the top 500 TFIIIC-bound genes with and without Pol III. We found TFIIIC-bound regions in H1 cells correlated positively with active chromatin marks (**Figure 9** **)**. As was previously reported in HeLa [7] and mouse ES cells [20], TFIIIC regions correlate well with CTCF binding sites in H1 cells (**Figure 9N**). Notably, most pluripotency-related transcription factors do not have high correlations with TFIIIC-only regions; however, we found that cMyc intersects at 59% of the regions (P<0.001, **Figure 9R** **)**.

## Discussion

This work provides the first genome-wide study of Pol III in human embryonic stem cells (hESCs), and addresses the relationship between Pol III occupancy, chromatin, and the pluripotency transcription network. To this end, we performed genome-wide ChIP-seq for Pol III and TFIIIC in H1 cells, and then compared our Pol III data with publicly available histone and transcription factor genome-wide maps to give us insights into Pol III localization.

Pol III is believed to bind opportunistically to its target genes; repressive chromatin prevents occupancy whereas open chromatin favors Pol III occupancy [5]–[8]. By several criteria, hESCs have a more open chromatin than differentiated cells; and in keeping with this notion, Pol III binds more target genes in H1 cells compared to other cell types. In H1 cells, more tRNAs are bound by Pol III, compared to HeLa cells. This observation also holds true in the induced pluripotent stem cell line as well (ADS-iPS). We also observed more Alu and MIR repeats bound by Pol III in these stem cell lines. At present, the functional consequence of such an increase in ES cells is unknown. It is possible that ES cells require more active tRNAs to support increased self renewal and growth. Alternatively, higher Pol III gene occupancy may simply reflect a more open chromatin state and increased opportunistic binding.

We examined the relationship of Pol III-bound regions with active chromatin in H1 cells. Pol III-bound regions (within 500 bp) in H1 cells correlated highly with active chromatin marks such as H3K4me3, H3K4me1, H3K4me2, H3K9ac, H3K27ac and H2AZ. The H3K4me3, H3K4me2, H3K9ac histone modifications, and H2AZ often mark Pol II promoters (defined as a 4 kb region flanking a known Pol II TSS), which is where about 20% of the Pol III-bound genes reside. H3K4me1 and H3K27Ac, on the other hand, mark more enhancer like chromatin, and about 80% of Pol III-bound genes localize to enhancers. Interestingly, both the promoter and enhancer class of Pol III-bound genes have Pol II, which typically peaks ∼300 bp upstream of the Pol III TSS. However, the correlation of Pol III with Pol II localization is ∼45% in H1, compared to 96% in HeLa cells [5]. This could be because of reduced reliance of Pol III on Pol II and its transcription machinery to create open chromatin in a more “open” ES cell genome.

Notably, Pol III-bound regions (within 500 bp) in H1 cells correlated with repressive marks: 24% with H3K9me3, and 40% with H3K27me3. This was in stark contrast with HeLa cells where there is no overlap of Pol III in regions with repressive marks (FDR 1%). This correlation in H1 cells with H3K27me3 could be because H3K27me3 often occurs in the context of bivalency. Analysis of Pol III-bound regions with regional H3K27me3 shows correlation with neighboring H3K4me3 (75%) and Pol II (44%). Incidentally, Pol II and H3K4me3 peak in between the H3K27me3 and Pol III peaks. These correlative results raised the possibility that the active chromatin created by Pol II insulates Pol III from repression by H3K27me3. In contrast, Pol III-bound regions with regional H3K9me3 correlate with H3K4me3 (13%) and Pol II (16%) only a moderate percentage of the time. However, it has been shown that H3K9me3 binding protein HP1 does not stably associate with chromatin until ES cells differentiate [16]. This lack of HP1 association with chromatin could insulate these genes from the normal consequences of H3K9me, keeping them open for Pol III binding to target genes.

Pol III localization in different cell types appears to be dependent on the cell type-specific chromatin landscape and cell type-specific transcription factors. In hESCs these cell- specific transcription factors include the pluripotency network factors, which were examined for binding correlations. Pol III-occupied regions in H1 cells correlated positively with the pluripotency factor NANOG 30% of the time, and to a lesser extent, though still statistically significant (P<0.001) with KLF4 (11%), OCT4 (10%), and SOX2 (9%). Previous Pol III genome-wide localization studies in mESCs did not address the relationship with pluripotency factors. Therefore, we analyzed mESC Pol III data with mouse pluripotency factor data. Pol III in mESC correlates positively with OCT4 at 36% (P<0.001) of regions and to a lesser extent with KLF4 (14%, P<0.001), NANOG (9%, P<0.001), SOX2 (8%, P<0.001) and cMYC (only 4%, P<0.001). These strong correlative results suggest that while pluripotency factors might generally help Pol III recruitment to target genes, the dependence on particular factors may vary across species. A limitation of our studies, inherent in studies of ES cells, is that we are not able to address whether these factors are functionally required for Pol III recruitment at these loci, as their knockdown leads to differentiation and major changes in chromatin structure. In human H1 cells, we also observed a high correlation with the zinc finger protein ZNF384 (32%, P<0.001). The function of ZNF384 is not well understood, but studies support roles in bone morphogenesis, by inhibiting BMP4 induced bone formation [34]. *Znf384−/−* mice have also been shown to have impaired spermatogenesis and fertility defects [35]. ZNF384 fusions to TAF15 and Ewing’s sarcoma gene EWSR1, caused by chromosome translocations have been implicated in acute leukemia [36], [37]. The role of ZNF384 in embryonic stem cells has not been explored, and its relationship with Pol III occupancy remains an interesting candidate for further study.

To understand how Pol III localization changes during changes in the chromatin landscape, we performed genome-wide Pol III analyses in iPSCs and precursor cells. When ADSCs are converted to ADS-iPSCs, Pol III binds more of its target genes; ADS-iPSCs gain 153 new Pol III-bound genes while losing only 10 (ADS-specific regions) Pol III-bound genes. The genes that gain Pol III are mildly correlated with pluripotency factors (data from H1 cells). These correlative results again conform to the notion that ES cells have a more open chromatin, and opportunistic Pol III binds its targets with assistance from the pluripotency network.

We also identified 40 Pol III genes that are bound in both ADSCs and ADS-iPSCs but not in H1 cells. We interpret this retention of Pol III binding as incomplete reprogramming of the Pol III repertoire. Why these particular loci are resistant to reprogramming is currently unknown, but may relate to the maintenance of adipose-specific transcription factors or chromatin states in the iPSCs. Thus, future applications of iPS technology should consider the completeness of Pol III gene reprogramming, in addition to chromatin and Pol II gene reprogramming, when evaluating reprogramming efficiency.

In keeping with our observations with tDNAs, we also observed more repeat elements (Alus and MIRs) occupied by Pol III in human stem cells. Recent bioinformatics and RNA-seq analyses revealed 24 Alu elements located adjacent to predicted miRNAs, raising the possibility that Pol III transcription of these Alu elements might enable the expression of the linked miRNAs downstream of the repeat [38] via a polycistronic model. However, our results do not support this notion, as we did not observe Pol III occupancy at any of these 60 Alu-miRNA pairs in our analysis. Furthermore, Alus have also been shown to be processed into repeat induced small RNAs (riRNAs) that regulate mRNA expression in ES cells and during retinoic acid mediated differentiation [39]. Differential localization of Pol III to these regulatory Alu repeats in ES versus differentiated cells is a possibility that needs to be explored.

ES cells have a prominent chromatin feature, bivalency, which transcriptionally poises Pol II genes. The repressive half of the bivalent mark, H3K27me3, is negatively correlated with Pol III binding in other cell types tested, whereas in ES cells Pol III occupies regions with flanking H3K27me3, but not when H3K27me3 directly overlaps the Pol III binding site. Interestingly, we observe a large number of instances where Pol II and H3K4me3 bind adjacent to the Pol III binding site, at which sites H3K27me3 plummets. Here, we speculate that the adjacent Pol II initiation and H3K4me3 modifications (and possibly other correlated modifications) deter H3K27me3, help facilitate nucleosome turnover, and provide an open binding site for Pol III. It is possible that TBP (in the context of TFIIIB) might be involved in recruiting Pol II to these regions to antagonize H3K27me3 spreading to the Pol III TSS. In contrast, in differentiated somatic cells, most (though not all) regions bearing H3K27me3 are simply silent, and not coincident with Pol II initiation or H3K4me3, and therefore deter Pol III. We note that beyond regulation of binding, studies in yeast and human cells strongly suggest a second axis for Pol III that involves regulation of activity via the sensing of the environment and cellular growth conditions; a property that involves the master regulator of Pol III, MAF1, which binds the large subunit of Pol III and at least one basal transcription factor [40]. Therefore the output of the Pol III system appears to involve two steps: 1) defining the Pol III gene repertoire, which is likely regulated by open chromatin, defined in part by the transcription factors residing in that cell (including pluripotency factors in ES cells), chromatin modifiers, and Pol II initiation, and 2) modulating the amount of transcription from the occupied repertoire loci, involving environmental and growth sensing via MAF1. Our results suggest that upon differentiation, Pol III binds fewer genes, likely owing to a transition to a more repressive chromatin state. We also notice that a fraction of the NANOG and Pol III bound genes are still bound by Pol III in differentiated cell types (which lack any pluripotency factors). This leads us to believe that upon differentiation of ES cells, the pluripotency factors may ‘hand off’ part of their responsibility for opening up chromatin for Pol III to an alternative set of transcription factors residing in that particular cell type, such as ETS1, c-JUN and c-FOS.

## Conclusions

This work provides the first examination of the RNA Pol III repertoire in human embryonic stem cells and induced pluripotent stem cells. We show that the Pol III repertoire in hESCs and iPSCs is larger than in differentiated cell types, owing to a more open chromatin status, compared to differentiated cells. Pol III also localizes to open/active chromatin in hESCs, in keeping with chromatin regulation of Pol III occupancy. Notably, we provide correlative evidence that the pluripotency factors in hESCs, in concert with active chromatin, may help Pol III bind its target genes. Interestingly, we observe a class of active Pol III-bound genes with neighboring repressive H3K27me3 mark. We observe at these active Pol III genes, a peak of H3K4me3 and/or RNA Pol II, between the H3K27me3 and Pol III binding peaks, suggesting that H3K4me3 and Pol II activity may “insulate” Pol III from neighboring repressive H3K27me3. Finally, we observe a small number of active Pol III genes that are common to ADSCs and ADS-iPSCs but not H1 cells, suggesting that the iPSCs retain a transcriptional memory of the active Pol III genes in their precursors.

## Data Access

Raw data for the study can be accessed at the SRA database using accession number SRP026209. Data tracks can be visualized at https://bioserver.hci.utah.edu/gnomex/gnomexFlex.jsp?topicNumber=11 using UCSC or IGV browsers.

## Supporting Information

Figure S1 **Quantification of ChIP Efficiency. A.** ChIP qPCR data showing relative Pol III enrichment at three known Pol III genes. 1∶100 of the eluate was used for the qPCR and the data was normalized to the Gene Desert region. **B.** ChIP qPCR data showing relative TFIIIC enrichment at tRNAs. 1∶100 of the eluate was used for the qPCR and the data was normalized to the Gene Desert region.
(TIF)

Figure S2 **H3K27me3, H3K4me3 and Pol II levels at bivalent Pol II and Pol III bound regions. A.** Box plot showing H3K27me3, H3K4me3 and Pol II levels at a Pol III-bound bivalent regions. H3K27me3, H3K4me3 and Pol II levels were calculated using DefinedRegionScanSeqs (program from USeq), 2 kb upstream of all Pol III-bound bivalent regions. The 2 kb region upstream was split into “2 kb to 500 bp upstream” and “−500 bp to TSS” revealing that H3K27me3 levels drop at “−500 to TSS” and H3K4me3 and Pol II levels rise in the same region. Asterisks indicate a significant P-Value <0.0001. **B.** Class average map of a bivalent Pol II promoter in H1 cells, showing H3K27me3, H3K4me3 and Pol II signal at an average bivalent Pol II promoter. X-axis indicates distance from the Pol II gene TSS and Y-axis shows normalized read counts for each factor.
(TIF)

File S1 **Supplemental Tables. 1.** Accession numbers of data analyzed in the paper. **2.** List of potential Annotated Pol III genes. **3.** Pol III bound regions in H1 cells with 1% FDR. **4.** TFIIIC bound top 500 regions in H1 cells. **5.** H1 specific Pol III bound genes (compared to HFF, HEK, HeLa). **6.** Pol III bound regions in ADS cells with 1% FDR. **7.** Pol III bound regions in ADSiPS cells with 1% FDR. **8.** Novel Pol III bound regions in H1 cells (at 1% FDR). **9.** Fraction Intersections between H1 Pol III and TFIIIC regions with chromatin marks and transcription factors.
(XLSX)

File S2 **Figure Methods.** Here we describe data analysis techniques used to generate the figures for the manuscript.
(DOCX)
