# Supplementary figures and images for: RNA Polymerase III Transcriptomes in Human Embryonic Stem Cells and Induced Pluripotent Stem Cells, and Relationships with Pluripotency Transcription Factors
Source: PLoS One. 2014 Jan 20;9(1):e85648. doi: 10.1371/journal.pone.0085648 (PMC3896398; doi:10.1371/journal.pone.0085648)

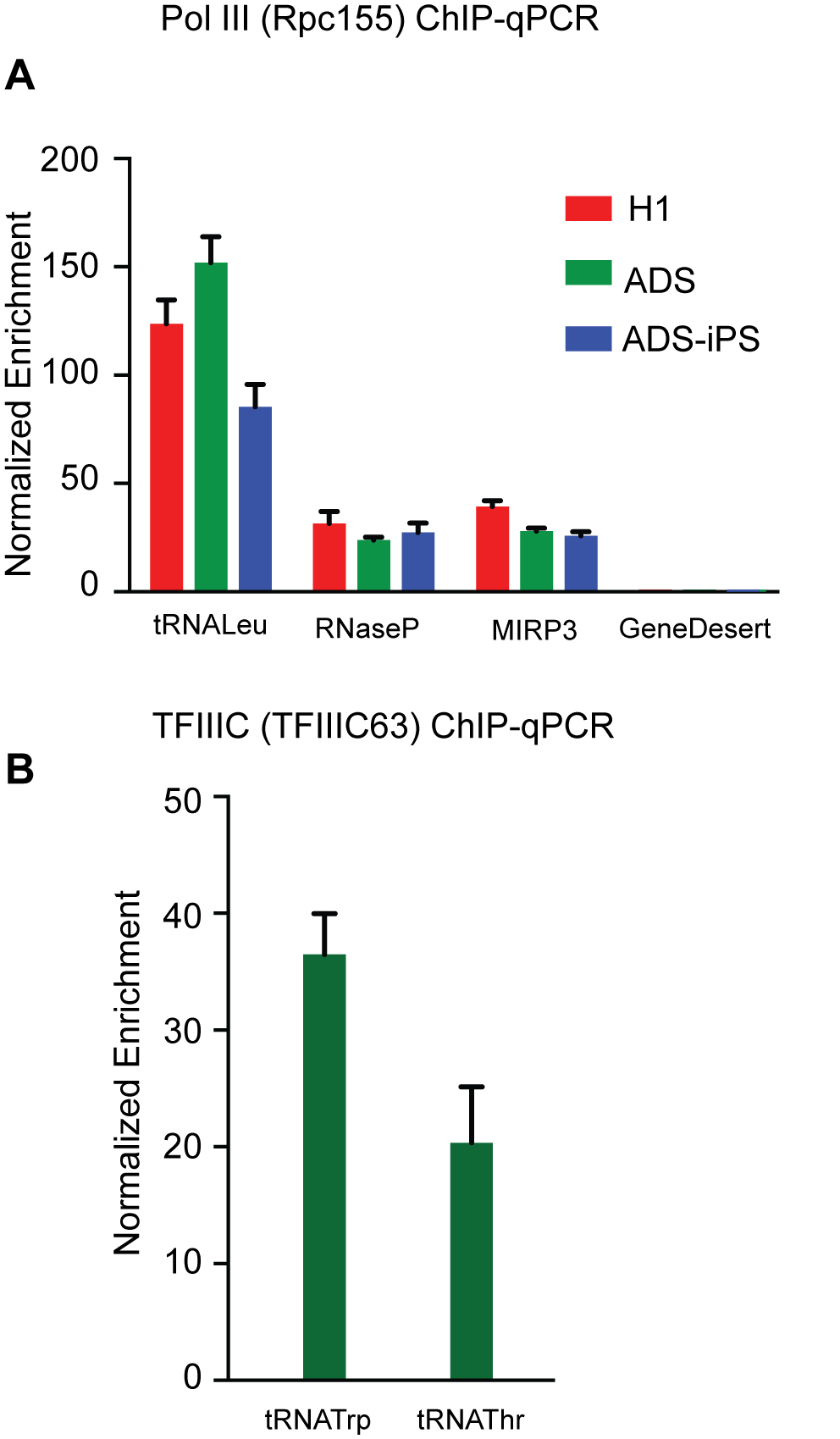

Supplement: Figure S1 — Quantification of ChIP Efficiency. A. ChIP qPCR data showing relative Pol III enrichment at three known Pol III genes. 1∶100 of the eluate was used for the qPCR and the data was normalized to the Gene Desert region. B. ChIP qPCR data showing relative TFIIIC enrichment at tRNAs. 1∶100 of the eluate was used for the qPCR and the data was normalized to the Gene Desert region. (TIF) [file pone.0085648.s001.tif]

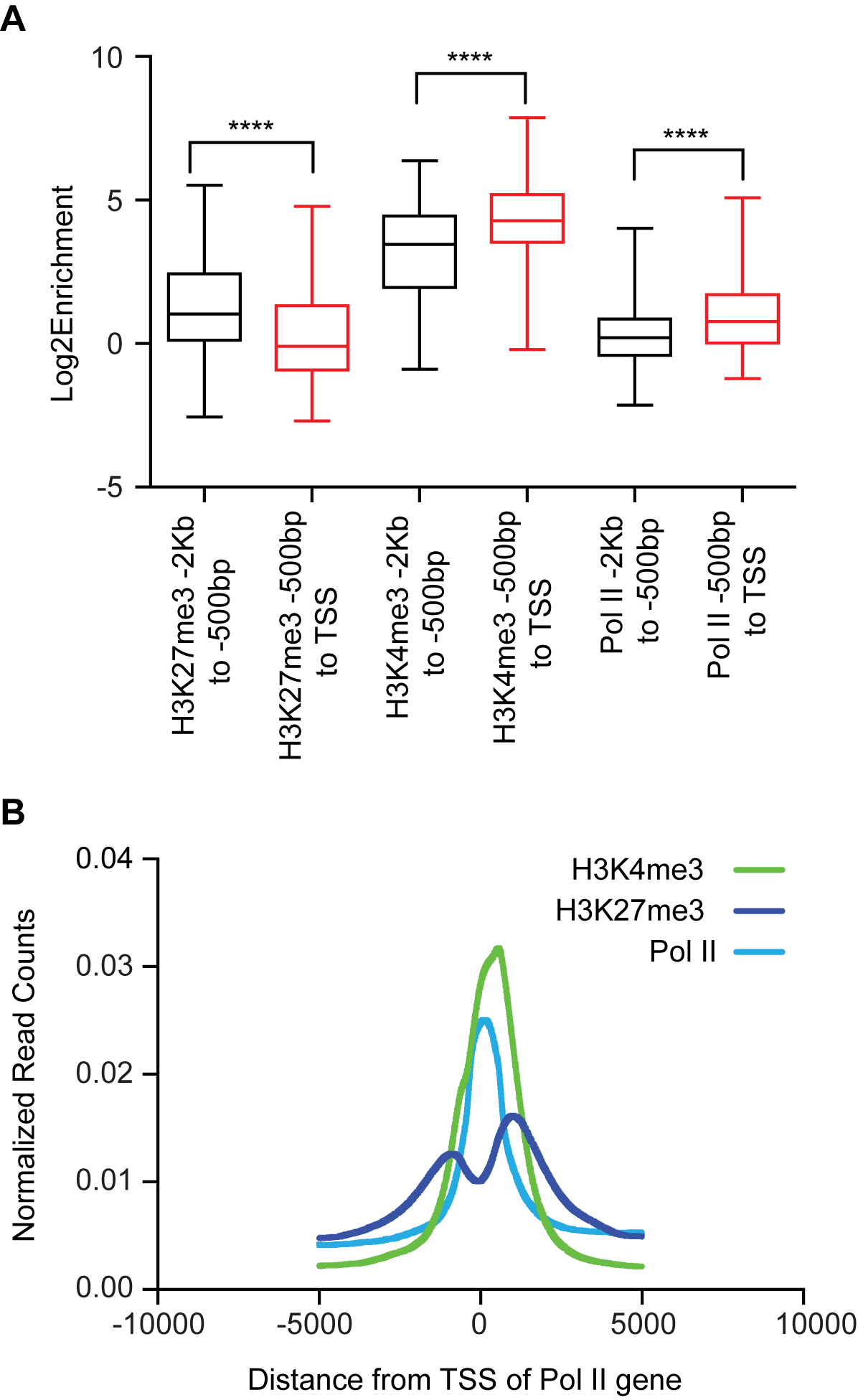

Supplement: Figure S2 — H3K27me3, H3K4me3 and Pol II levels at bivalent Pol II and Pol III bound regions. A. Box plot showing H3K27me3, H3K4me3 and Pol II levels at a Pol III-bound bivalent regions. H3K27me3, H3K4me3 and Pol II levels were calculated using DefinedRegionScanSeqs (program from USeq), 2 kb upstream of all Pol III-bound bivalent regions. The 2 kb region upstream was split into “2 kb to 500 bp upstream” and “−500 bp to TSS” revealing that H3K27me3 levels drop at “−500 to TSS” and H3K4me3 and Pol II levels rise in the same region. Asterisks indicate a significant P-Value <0.0001. B. Class average map of a bivalent Pol II promoter in H1 cells, showing H3K27me3, H3K4me3 and Pol II signal at an average bivalent Pol II promoter. X-axis indicates distance from the Pol II gene TSS and Y-axis shows normalized read counts for each factor. (TIF) [file pone.0085648.s002.tif]
