# Supplementary material for: RNA Polymerase III Transcriptomes in Human Embryonic Stem Cells and Induced Pluripotent Stem Cells, and Relationships with Pluripotency Transcription Factors
Source: PLoS One. 2014 Jan 20;9(1):e85648. doi: 10.1371/journal.pone.0085648 (PMC3896398; doi:10.1371/journal.pone.0085648)
Supplement: File S2 — Figure Methods. Here we describe data analysis techniques used to generate the figures for the manuscript. (DOCX) [file pone.0085648.s004.docx]

**Supplementary Information**

**RNA Polymerase III transcriptomes in human embryonic stem cells and induced pluripotent stem cells, and relationships with pluripotency transcription factors**

**Ravi K. Alla^1,2^ and Bradley R. Cairns^1,2,3^**

^1^Department of Oncological Sciences, Huntsman Cancer Institute, University of Utah School of Medicine, Salt Lake City, UT, USA.

^2^Howard Hughes Medical Institute, Chevy Chase, MD, USA.

^3^Correspondence: brad.cairns@hci.utah.edu

**Figure Methods**

**Figure 2**

**A,C,D,E.** Pol III, TFIIIC and small RNA data for H1 cells was analyzed as described in the methods section. The snapshot shows Pol III, TFIIIC QValFDR data and the small RNA read coverage data, as visualized in the IGB genome browser.

**B.** Enriched regions for Pol III and TFIIIC were individually intersected (using IntersectRegions program from USeq with options -g 50 -n 1000) with a list of all known, annotated Pol III genes. This list of Pol III genes, has all the tRNAs, 5S rRNAs, SNARs, 7SL, 7SK, HVG, HY RNAs, BC200, other known Pol III transcribed genes and all the Alus and MIRs from the repeatmasker track from UCSC hg18 genome build. Each gene name in this list was then made unique by appending a line number to it. Once Pol III-bound and TFIIIC-bound genes were identified, Venn diagram generator from Chris Seidel (http://www.pangloss.com/seidel/Protocols/Venn.cgi) was used to perform simple Venn intersections. The resulting intersection numbers from the Venn diagram generator were plugged into the eulerAPE program (http://www.eulerdiagrams.org/eulerAPE/) to generate area-proportional Venn diagrams for visualization. The intersections are statistically significant with a p<0.001 as ascertained using IntersectRegions.

**Figure 3**

**A.** The Pol III-bound regions were intersected with the list of previously mentioned Pol III annotated genes. Venn Intersections were performed as in Figure 1E and 4-way intersections were plotted using the R package VennDiagram[1].

**B-G.** QValFDR tracks for HeLa and HEK293 cell lines were obtained from previous studies in our lab[2] and visualized along with H1 data on IGB at different genomic loci.

**Figure 4**

To generate these class average maps, Pol III-bound tRNAs genes were ranked according to their Pol III QValFDR values. Then 3 different region files, top 50 (blue), middle 50 (red) and bottom 50 (yellow) tRNA genes that were bound by Pol III were made. tRNAs genes that were not bound by either Pol III or TFIIIC were used as a negative control (green). These regions were then extended 2kb up and downstream of the tRNA TSS to give a 4kb bed file. Then USeq[3] program AggregatePlotter was run (with options -r -n -u 1) with these 4 bed files and point data for each of the different histone marks and transcription factors. The output is normalized read count, normalized for number of regions in each bed file. The output was transferred to Prism for graphing. The intersection fractions were calculated using IntersectRegions, (with options -g 500 -n 1000) using all Pol III-bound regions as the first file and chromatin marks/transcription factor regions as the second file. The intersection fractions are significant, with p<0.001

**Figure 5**

**A-D.** Pol III-bound regions were intersected with H3K27me3 regions (using IntersectRegions with options -g 1000 -n 1000) to give a list of Pol III regions that had the H3K27me3 mark. Example loci were then picked from this intersected list and visualized on IGB.

**E.** To analyze H3K27me3 levels at Pol III-bound and unbound tRNAs in H1 and HeLa cells, 4kb regions (as above) and 500bp regions flanking the tRNA TSSs were used. DefinedRegionScanSeqs program was then run on the Pol III-bound and unbound tRNA regions in both cell types, using the H3K27me3 point data for each cell type. The resulting log2enrichment scores were imported to Prism for box plot and t-test analysis.

**F.** Pol III-bound regions marked by H3K27me3 were isolated as explained above. These regions were then intersected with Pol III annotated gene list. The resulting gene bed file was extended 2kb up and downstream. AggregatePlotter was then run on this bed file with H3K4me3, H3K27me3, Pol II, Pol III and CTCF point data from H1 cells. The resulting data was then graphed in Prism.

**Figure 6**

**A.** For this intersection analysis, pybedtools[4] was used. This program takes 3 bed files and outputs intersection values. IntersectRegions shows these intersections to be statistically significant. eulerApe program was used to generate the area proportional Venn diagrams.

**B-D.** Class average maps for NANOG, OCT4 and SOX2 were generated as described in Figure 3.

**E-H.** Pol III-bound regions were intersected with NANOG-bound regions in H1 cells (using IntersectRegions -g 500 -n 1000). Example loci from the intersected lists were visualized in IGB.

**Figure 7**

To generate these class average maps, Pol III-bound genes in mouse ES cells [5] were used. tRNAs genes in this list were excluded from UCSC mm9 tRNA track, to give an unbound (by Pol III) tDNA bed file. These two bed files were subject to AggregatePlotter (with options -r -n -u 1) using mouse ES cell point data for OCT4, NANOG, KLF4, SOX2, cMYC, SUZ12, ZC3H11A and ZNF384. IntersectRegions (-g 500 -n 1000) was run using Pol III-bound genes as the first file and transcription factor regions as second file, give intersection fractions (with P< 0.001).

**Figure 8**

**A.** Pol III-bound regions in H1, ADS and ADS-iPS cells were intersected with Pol III annotated gene list to obtain bound Pol III genes in each cell type. Intersection analysis was then performed on these 3 gene lists as described in Figure 1E. eulerAPE was used to create the area proportional Venn diagram.

**B-E.** To identify novel Pol III regions in H1 cells, Pol III-bound region file was intersected with the Pol III annotated gene list, to generate a difference list. Examples from this list were visualized on IGB. The small RNA data plotted is the read coverage track for H1 and H9 cells.

**Figure 9**

Top 500 bound TFIIIC regions in H1 cells were first intersected with Pol III annotated gene list, excluding Alus and MIRs (IntersectRegions -g 50 -n 1000), followed by intersection with Pol III-bound regions (-g 100 -n 1000). This analysis gave two bed files; one gene set that was bound by both TFIIIC and Pol III, the other with only TFIIIC (without Pol III). These two lists were then used in AggregatePlotter, normalized for number of regions in each of the bed files (with options -r -n -u 1) and the result graphed in Prism.

**References**

1. Chen H, Boutros PC (2011) VennDiagram: a package for the generation of highly-customizable Venn and Euler diagrams in R. BMC Bioinformatics 12: 35. doi:10.1186/1471-2105-12-35.

2. Oler AJ, Alla RK, Roberts DN, Wong A, Hollenhorst PC, et al. (2010) Human RNA polymerase III transcriptomes and relationships to Pol II promoter chromatin and enhancer-binding factors. Nat Struct Mol Biol 17: 620–628. doi:10.1038/nsmb.1801.

3. Nix DA, Courdy SJ, Boucher KM (2008) Empirical methods for controlling false positives and estimating confidence in ChIP-Seq peaks. BMC Bioinformatics 9: 523. doi:10.1186/1471-2105-9-523.

4. Dale RK, Pedersen BS, Quinlan AR (2011) Pybedtools: a flexible Python library for manipulating genomic datasets and annotations. Bioinformatics 27: 3423–3424. doi:10.1093/bioinformatics/btr539.

5. Carrière L, Graziani S, Alibert O, Ghavi-Helm Y, Boussouar F, et al. (2011) Genomic binding of Pol III transcription machinery and relationship with TFIIS transcription factor distribution in mouse embryonic stem cells. Nucleic Acids Res 40: 270–283. doi:10.1093/nar/gkr737.
